# Cholesterol contributes to dopamine-neuronal loss in MPTP mouse model of Parkinson’s disease: Involvement of mitochondrial dysfunctions and oxidative stress

**DOI:** 10.1371/journal.pone.0171285

**Published:** 2017-02-07

**Authors:** Rajib Paul, Amarendranath Choudhury, Sanjeev Kumar, Anirudha Giri, Rajat Sandhir, Anupom Borah

**Affiliations:** 1 Cellular and Molecular Neurobiology Laboratory, Department of Life Science and Bioinformatics, Assam University, Silchar, Assam, India; 2 Microbial and Molecular Immunology Laboratory, Department of Life Science and Bioinformatics, Assam University, Silchar, Assam, India; 3 Environmental Toxicology Laboratory, Department of Life Science and Bioinformatics, Assam University, Silchar, India; 4 Department of Biochemistry, Panjab University, Chandigarh, India; Emory University, UNITED STATES

## Abstract

Hypercholesterolemia is a known contributor to the pathogenesis of Alzheimer’s disease while its role in the occurrence of Parkinson’s disease (PD) is only conjecture and far from conclusive. Altered antioxidant homeostasis and mitochondrial functions are the key mechanisms in loss of dopaminergic neurons in the substantia nigra (SN) region of the midbrain in PD. Hypercholesterolemia is reported to cause oxidative stress and mitochondrial dysfunctions in the cortex and hippocampus regions of the brain in rodents. However, the impact of hypercholesterolemia on the midbrain dopaminergic neurons in animal models of PD remains elusive. We tested the hypothesis that hypercholesterolemia in MPTP model of PD would potentiate dopaminergic neuron loss in SN by disrupting mitochondrial functions and antioxidant homeostasis. It is evident from the present study that hypercholesterolemia in naïve animals caused dopamine neuronal loss in SN with subsequent reduction in striatal dopamine levels producing motor impairment. Moreover, in the MPTP model of PD, hypercholesterolemia exacerbated MPTP-induced reduction of striatal dopamine as well as dopaminergic neurons in SN with motor behavioral depreciation. Activity of mitochondrial complexes, mainly complex-I and III, was impaired severely in the nigrostriatal pathway of hypercholesterolemic animals treated with MPTP. Hypercholesterolemia caused oxidative stress in the nigrostriatal pathway with increased generation of hydroxyl radicals and enhanced activity of antioxidant enzymes, which were further aggravated in the hypercholesterolemic mice with Parkinsonism. In conclusion, our findings provide evidence of increased vulnerability of the midbrain dopaminergic neurons in PD with hypercholesterolemia.

## Introduction

Parkinson’s disease (PD), the most common neurodegenerative movement disorder, occurs due to dopaminergic neurodegeneration in substantia nigra (SN) pars compacta region of the midbrain, thereby leading to depletion in the levels of dopamine in striatum [1,2]. Clinical diagnostic features of PD include the presence of Lewy bodies in brain formed mainly by the α-synuclein protein aggregates [3] and the cardinal motor behavioral abnormalities: bradykinesia, tremor at rest, postural instability and rigidity [2,4]. Among the different mechanisms that are reported to cause death of midbrain dopamine neurons in PD, mitochondrial dysfunction with compromised antioxidant support system is undebatable [5–7]. Many endogenous molecules, such as homocysteine, phenethylamine and dopamine itself have been implicated in the pathogenesis of PD [8–10]. Interestingly, cholesterol and/or its oxidized metabolites (oxysterols) have been suggested to be putative endogenous contributors towards the appearance of Parkinsonian pathologies [11,12]. Clinical studies suggest a strong link between elevated levels of plasma cholesterol and incidence of PD [13, 14]. Moreover, high-fat diet exacerbates Parkinsonian pathologies, including loss of dopamine-neurons in animal models of PD [15,16]. Several findings have shown the occurrence of pathologies in cellular neuronal models caused by excess exposure to cholesterol or oxysterols which are equivalent to PD [17–19]. Reduced activity of mitochondrial complex-I and antioxidant enzymes, as well as depleted levels of the antioxidant molecule (reduced glutathione, GSH) have been reported in non-dopaminergic regions of brain of hypercholesterolemic animals [20–23]. However, the role of hypercholesterolemia on the functional status of midbrain dopamine neurons in PD brain remains inconclusive. Thus, we have investigated whether hypercholesterolemia would deteriorate dopaminergic neurodegeneration in the light of functional impairment mitochondrial complexes and oxidative stress in animal model of PD.

## Materials and methods

### Animals

Eight weeks old male Swiss albino mice (21–22 g) were used in the present study. The animals were maintained under standard laboratory conditions of temperature (24 ± 2°C) and humidity (60 ± 5%). They were provided with food and water *ad libitum*.

### Chemicals

1-methyl-4-phenyl-1,2,3,6-tetrahydropyridine hydrochloride (MPTP; M0896), dopamine (H8502), 2,3-dihydroxybenzoic acid (2,3-DHBA; 126209), 2,5-dihydroxybenzoic acid (2,5-DHBA; 78069), reduced glutathione (GSH; G4251), coenzyme Q_0_, ethylenediaminetetraacetic acid disodium salt (EDTA), heptane sulfonic acid, triethylamine, orthophosphoric acid, chloral hydrate, poly-L-lysine, perchloric acid (HClO_4_; 380083) hydrogen peroxide (H_2_O_2;_ V800211), Triton X-100, 3,3-diaminobenzidine (DAB) liquid substrate system (D3939) and water for high-performance liquid chromatography (HPLC; V800443) were purchased from Sigma-Aldrich Co (St. Louis, MO, USA). Cholesterol (97900), acetonitrile, nitro blue tetrazolium (NBT; 48898), cytochrome c (oxidized; 81551), pyrogallol, salicylic acid, glycerol jelly and paraformaldehyde (PFA) were obtained from SISCO Research Laboratories (Mumbai, India). Rabbit anti-tyrosine hydroxylase (TH) antibody (ab112) and donkey serum (ab7475) was purchased from Abcam (Cambridge, UK). Anti-rabbit goat secondary antibody tagged with horseradish peroxidase (HRP; ap307p) was purchased Millipore Co. (USA).

### Experimental design

**Fig 1:**
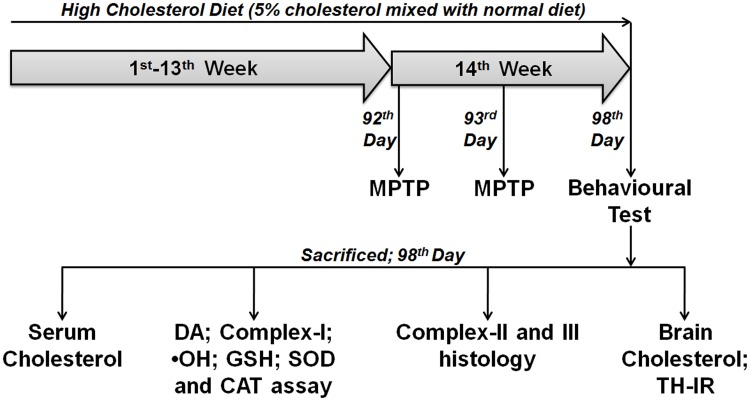
Schematic representation of the experimental paradigm. [Abbreviations: DA, dopamine; •OH, hydroxyl radical; GSH, reduced glutathione; SOD, superoxide dismutase; CAT, catalase; TH-IR, tyrosine hydroxylase-immunoreactivity].

Mice were initially divided into two groups: control group (given normal rodent chow) and high cholesterol diet group (HCD; given cholesterol at the dose of 5% w/w mixed with normal rodent chow). Both groups of mice received equal calories of food throughout the experimental period. At the end of 13-weeks, a group of control and HCD mice were injected with MPTP (30 mg/kg b.w.; i.p.) for two consecutive days to induce PD. Thus, there were four groups: control, HCD, MPTP, and HCD+MPTP. On the 7^th^ day following the first dose of MPTP, all groups of mice were subjected to motor behavioral tests (akinesia, catalepsy, and swim test), following which the mice was sacrificed for the neurochemical and histochemical studies. Measuring the serum total cholesterol levels at the end of treatment window confirmed hypercholesterolemia. Mice were decapitated for analysis of dopamine, complex-I, and oxidative stress parameters (hydroxyl radical, GSH, SOD and catalase) in the brain. Mice were either perfused with 10% glycerol for histoenzymological studies (mitochondrial complex-II and III) or 4% PFA for cholesterol histology (liver and brain) and TH-immunoreactivity (Fig 1).

### Serum total cholesterol

At the end of the feeding period (98^th^ day), mice were anesthetized with chloral hydrate (350 mg/kg; i.p.) and blood was collected by cardiac puncture. The serum was separated by centrifugation at 3000 x g for 4 minutes. The serum total cholesterol was estimated by *in vitro* enzymatic method using a colorimetric kit (CHOL, Autopak, Siemens) following the manufacturer’s instructions. The concentration of cholesterol in serum is directly proportional to the intensity of the red complex generated, the absorbance of which was recorded at 500 nm using Spectrophotometer [24].

### Liver and brain cholesterol histochemistry

Liver and brain (striatum) cholesterol was measured by Schultz’s method. The PFA (4%) fixed liver and brain tissues were subjected to cryocut using cryostat (0620E Cryostat, Thermo Shandon, UK) and coronal sections of the liver and brain (mainly striatum) were taken. 20 μm sections were incubated at room temperature in alum of iron (ferrous aluminium sulfate) for 5 days and then one drop of freshly prepared acid mixture (1:1, glacial acetic acid and sulphuric acid) was poured over the sections. The principle of this method deals with the Liebermann-Burchard reaction which gives greenish blue color to cholesterol and enables proper quantification for the presence of cholesterol in tissue [25]. The optical density of the characteristic greenish blue color was measured using Fiji Version of ImageJ software [26].

### Analysis of behavioral parameters

#### Akinesia

Akinesia was measured by noting the latency in second (s) of the animals to move all four limbs and the test was terminated if the latency exceeded 180 s. Each animal was initially acclimatized for 5 minutes on an elevated wooden platform (40 cm×40 cm× 30 cm). Using a stopwatch, the time taken by the animal to move all the four limbs was recorded. This exercise was repeated 5 times for each animal [27].

#### Catalepsy

The inability of an animal to correct an externally imposed posture, was measured by placing the animals on a flat horizontal surface with both the hind limbs on a square wooden block (3 cm high) and the latency in seconds was measured to move the hind limbs from the block to the ground [27].

#### Swim test

Swimming ability test was carried out in tubs with 12 cm high water maintained at 27 ± 2°C. The animals were placed in water and the swimming ability for a period of 10 minutes was scored every minute as: 3-continuous swimming, 2-swimming with occasional floating, 1- more floating with occasional swimming with hind limbs, and 0-hind part sinks with only the head floating, following the procedure described earlier [27,28].

### Striatal dopamine levels

To investigate the effect of hypercholesterolemia on striatal dopamine level in Parkinsonian animals, mice were sacrificed by decapitation on the last day of treatment. Whole brain was removed from the calvarium and the nucleus caudatus putamen (NCP, striatum) dissected out. After sonication in ice-cold 0.1 M HClO_4_, containing 0.01% EDTA, the tissues were centrifuged for 5 min at 10,000 x g 10 μL supernatant was injected into the HPLC system equipped with the Electro-Chemical detector (HPLC-ECD; Waters, Austria) at a flow rate 0.8 ml/min. The electrochemical detection was performed at + 740 mV. The composition of the mobile phase was 8.65 mM heptane sulfonic acid, 0.27 mM EDTA, 13% acetonitrile, 0.43% triethylamine and 0.22% orthophosphoric acid, as reported earlier [29].

### Tyrosine hydroxylase immunohistology

Mice were anesthetized with chloral hydrate (350 mg/kg; i.p.) and perfused intracardially with phosphate buffered saline (PBS, 0.1 M; pH 7.4) followed by 4% w/v PFA in PBS. Brains were removed and kept overnight in the same fixative, transferred to 30% w/v sucrose solution. 20 μm thick coronal sections passing through the NCP and SN were taken using Cryotome in poly-L-lysine coated slides. The sections were rinsed three times with 0.1 M Tris-buffered saline (TBS, 0.1 M; pH 7.4), incubated in 3% H_2_O_2_ in TBS, permeabilized with 0.3% Triton X-100, and blocked with 10% donkey serum containing 0.3% Triton X-100. The sections were incubated overnight with primary antibody for TH (1:700) in TBS, containing 2% donkey serum at 4°C and then incubated with HRP-conjugated secondary antibody (1:1000) in TBS for 1 h at room temperature. Colour development was performed by incubating the sections in DAB-liquid substrate system and then sections were washed, dehydrated, cleared in xylene, mounted in DPX and photographed under bright field illumination using a digital SLR camera attached to the Trinocular microscope (Eclipse Ci-L, Nikon, Japan) [30].

### Mitochondrial complex-I activity

Mitochondrial complex-I activity was assayed as described earlier [31]. Animals were sacrificed by decapitation, brains were removed immediately and the NCP and SN regions of brain were either micropunched or dissected out from fresh frozen sections of 1 mm thick. The tissues were sonicated in 0.1 M potassium phosphate buffer (pH 7.8), centrifuge at 600 × g for 50 s and complex-I activity was assayed in the supernatant. The reaction mixture containing mitochondrial fractions (100 μg), sodium azide (5 mM), coenzyme Q_0_ (50 mM) and potassium phosphate buffer (10 mM; pH 7.2) was incubated at 32°C for 3 min. The reaction was initiated by addition of NADH (100 μM) in the reaction mixture and the rate of decrease in the absorbance was monitored at 340 nm for 3 min.

### Mitochondrial complex-II and complex-III activity

Mice were anesthetized with chloral hydrate (350 mg/kg; i.p.) and perfused intracardially with PBS followed by 10% (50 ml) ice cold glycerol. Brains were removed and kept overnight in 30% sucrose. 20 μm thick coronal sections passing through the NCP and SN were cut using Cryotome and transferred to poly-L-lysine coated slides. The optical density of the characteristic stain intensity was measured using Fiji Version of ImageJ software [26].

The sections from the intended regions of brain were incubated in PBS (0.1 M; pH 7.4) at 37°C for 10 min to activate the enzyme and then incubated in dark at 37°C for 35 min [32]. The reaction mixture contains 30 mM NBT, 50 mM sodium succinate in 50 mM potassium phosphate buffer (pH 7.4). After incubation, sections were gently washed with the reaction buffer, mounted in glycerol and photographed immediately.

Mitochondrial complex-III histochemistry was performed in NCP and SN regions of brain, following Govindaiah et al. [33]. The sections were rinsed three times in PBS (0.1 M; pH 7.4) and incubated in the reaction mixture of 30 ml containing DAB (17 mg), cytochrome c (7 mg) and sucrose (1.5 g) in PBS (0.1 M; pH 7.4) at 37°C for 50 min. After the incubation, the sections were rinsed gently with PBS, dried, mounted with DPX and photographed.

### Estimation of hydroxyl radical

The adducts of salicylic acid, 2,3- and 2,5-DHBA, were estimated using HPLC-ECD system to quantify the amount of hydroxyl radical (•OH) generated in brain of animals [34]. On the last day of treatment period (98^th^ day), different groups of animals were injected with salicylic acid (100 mg/kg; i.p) and sacrificed by decapitation 2 h after the injection. The brains were quickly removed from calvarium; the NCP separated out and SN micropunched from 1 mm frozen sections, processed with ice-cold HClO_4_ (0.1 M) containing 0.01% EDTA and centrifuged at 10,000 × g for 5 min. The supernatant (10 μL) was injected into the HPLC-ECD system to measure the levels of 2,3- and 2,5-DHBA against the standards [34]. The mobile phase composition was same as that was used for the detection of dopamine.

### Reduced glutathione levels

Animals from different groups were sacrificed on the last day of treatment (98^th^ day) and the level of reduced glutathione (GSH) was assayed from NCP and SN by employing an HPLC-ECD procedure [35] with minor modification. The tissues were sonicated in 10 volumes of deionized water and 20 μl of tissue homogenate mixed with a solution containing 1.4 mM sodium borohydride, 1.5 mM EDTA, 66 mM sodium hydroxide and 10 μL of n-amyl alcohol, and incubated at 40°C in a water bath for 30 min. Proteins were precipitated by addition of 25 μl of ice-cold 0.4 M HClO_4_ and separated after centrifugation at 14,000 x g for 25 min at 4°C. The supernatant (10 μl) was injected into the HPLC-ECD system. The mobile phase consisted of 50 mM sodium phosphate monobasic, 1.0 mM 1-octanesulfonic acid, and 2% acetonitrile (v/v), and the pH adjusted to 2.7 with 85% ortho-phosphoric acid. The flow rate was 0.8 ml/min and the electrochemical detection was performed at +850 mV.

### SOD activity

Mice were sacrificed on the 7^th^ day after the first dose of MPTP to the respective groups. SOD activity was analyzed in the cytosolic fractions of NCP and SN, which represent Cu/Zn-SOD, following the method as described earlier [36] with slight modification [37]. The assay mixture (3 ml) contained 0.2 mM of pyrogallol, 1.5 mM of EDTA and 50 mM of Tris-HCl buffer (pH 8.2). Auto-oxidation of pyrogallol was measured using spectrophotometer at 420 nm for 3 min with or without the enzyme. The inhibition of pyrogallol oxidation was linear with the activity of the enzyme present. Fifty percent inhibition in pyrogallol auto-oxidation/mg protein/min is taken as one unit of the enzyme activity.

### Catalase activity

Catalase activity was assayed based on the method described earlier [38]. Mice were sacrificed on the 7^th^ day after the first dose of MPTP and the catalase activity was analyzed in the cytosolic fractions of NCP and SN. The assay mixture of 1 ml contained suitably diluted protein (150 μg), in 50 mM of phosphate buffer (pH 7.0). The reaction was started by the addition of H_2_O_2_ (30 mM) and decay of H_2_O_2_ was measured at 240 nm for 30 s in presence of the enzyme in the spectrophotometer. The specific activity is represented as change in absorbance/min/mg protein.

### Tyrosine hydroxylase-positive neuronal count

Tyrosine hydroxylase-positive nigral dopaminergic neurons were counted using ImageJ (Fiji version) software [39]. The first section was chosen randomly and thereafter every sixth section was selected through the entire substantia nigra from control and treated groups [40]. Cell counting parameters were optimized based on a pilot study in both control and treated animals. Sections from five different brain samples were considered for each group.

### Ethical statement

The experimental protocols used in the present study have specifically been approved by the Animal Ethics Committee, Assam University, Silchar, India.

### Statistical analysis

The data were analyzed employing Student’s t-test and one-way ANOVA with necessary post-hoc test. Results are given as mean ± S.E.M. Values of p≤ 0.05 were considered significant. GraphPad Prism version 7.0 software was used for statistical analysis.

## Results

### High cholesterol diet causes hypercholesterolemia

**Fig 2:**
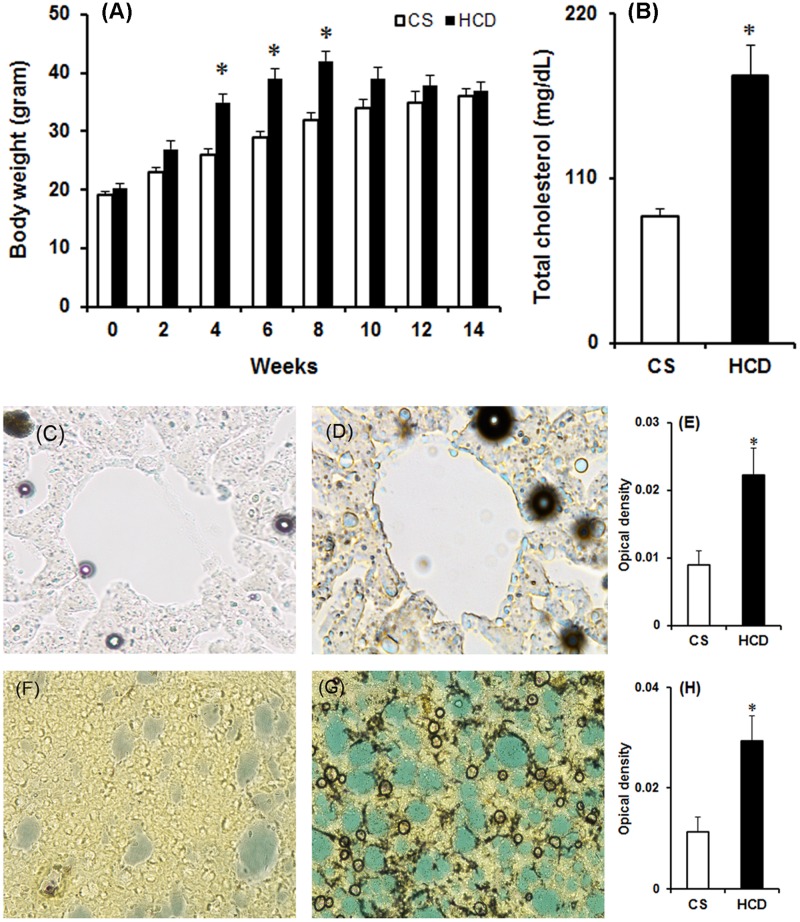
Effect of high cholesterol diet on (A) body weight, (B) serum total cholesterol, and cholesterol level in (C-D) liver and (F-G) brain (striatum). Mice were fed with high cholesterol diet (HCD) or standard diet (control, CS) for 14 weeks. Body weight (in gram) was measured two weeks apart from the start of treatment till 14^th^ weeks. On the last day of diet, blood was collected by cardio-punctured method and serum total cholesterol was estimated by enzymatic method. Accumulation of cholesterol in liver and striatum region of brain was estimated from fixed tissues by Schultz’s method. Optical density of characteristic greenish-blue colour for cholesterol in liver (E) and striatum (H) was measured using ImageJ software. The results given are mean ± S.E.M. *p ≤0.05 as compared to CS (n = 8).

Analyzing the serum and tissue total cholesterol levels confirmed hypercholesterolemic in mouse. The serum levels of total cholesterol in mice subjected to HCD were elevated significantly (2.1-fold) compared to the mice provided with normal rodent chow (179.23±16.45 vs. 85.4±7.2 mg/dL; Fig 2B). Schultz’s method was used to analyze the tissue levels of cholesterol in liver and NCP region of brain of the animals provided with HCD. Histochemical estimation revealed a marked increase of cholesterol levels in liver (Fig 2D) and NCP region of brain (Fig 2G) of HCD animals as compared to the control. The optical density of the blue or blue-green colour, that indicates cholesterol content in tissues, was estimated from serial sections of control and HCD animals (n = 5) which showed a significant increase in cholesterol content by 2-fold in liver (Fig 2E) and 2.5-fold in NCP (Fig 2H) of HCD animals. There was a trend of increase in body weight of HCD animals which significantly increased from 4^th^ to 8^th^ weeks of feeding following which a non-significant decrease was observed as compared to control animals (Fig 2A). The body weight increased by 15.5%, 21.5% and 22% respectively on 4^th^, 6^th^ and 8^th^ weeks in HCD animals. The body weight of mice maintained on the normal diet has increased gradually during the treatment period.

### Hypercholesterolemia on Parkinsonian motor behavior

**Fig 3:**
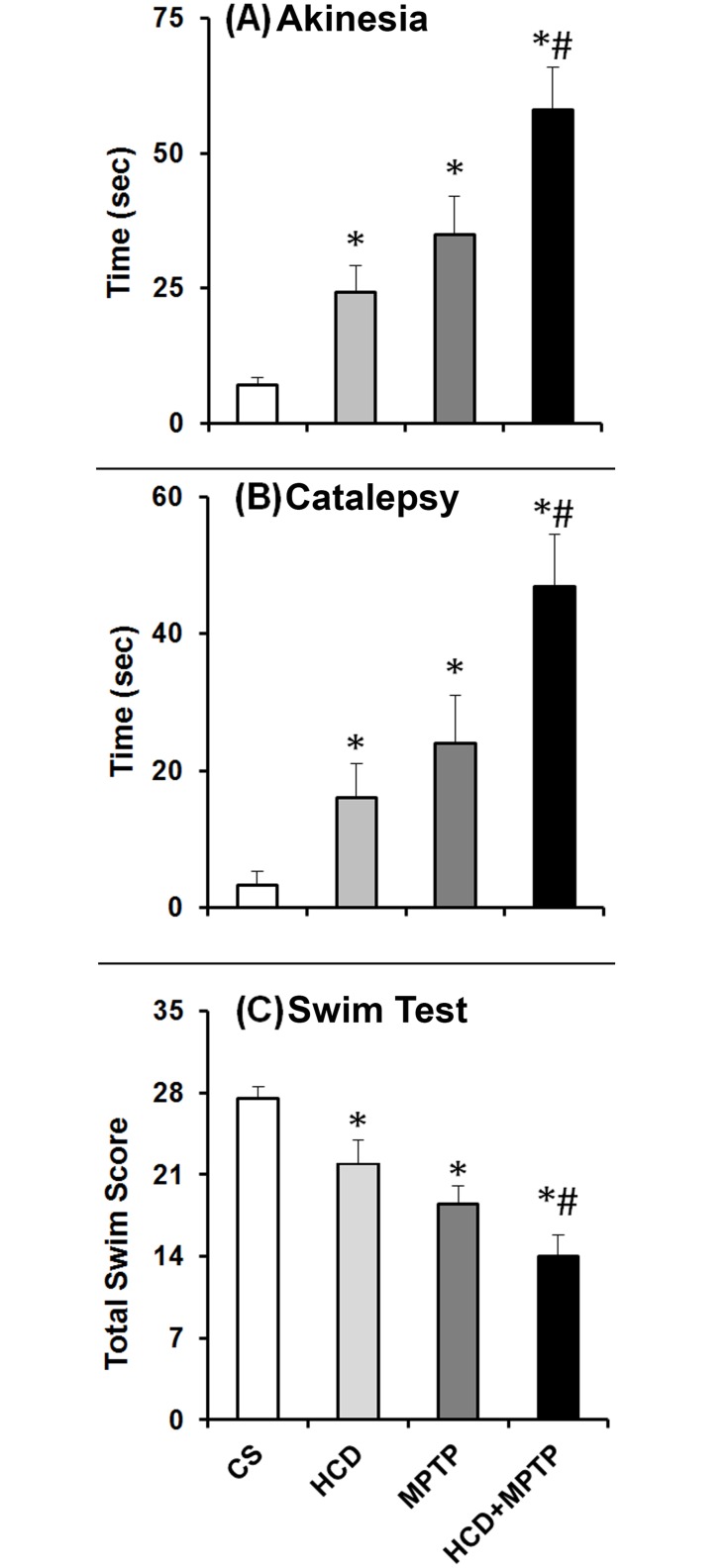
Effect of hypercholesterolemia on Parkinsonian motor behavior. In the last week of the 14 week treatment period, all the groups of animals were tested for (A) akinesia (B) catalepsy and (C) Swim test. The results are given as mean ± S.E.M. *p ≤ 0.05 as compared to control (CS) and #p ≤ 0.05 as compared to MPTP alone treated group (n = 6).

Administration of MPTP caused significant motor behavioral abnormalities in mice when tested on the 7^th^ day following the first dose of MPTP (Fig 3). As compared to control, the akinesia and catalepsy scores were increased by 5- and 7.4-fold respectively, while the total swim score was decreased by 32.7% in MPTP-treated mice. Similarly, HCD animals were found to be akinetic as well as cataleptic and exhibited poorer swimming ability as compared to control. Interestingly, administration of MPTP (30 mg/kg b.w.; two consecutive days) in HCD mice caused significant decrease in the tested motor behavioral scores as compared to MPTP alone treated mice. In HCD+MPTP treated animals, there occurred 1.66- and 1.96-fold increase in akinesia and catalepsy scores respectively, while swimming score was decreased by 24.32% compared to MPTP alone treated animals (Fig 3).

### Hypercholesterolemia on striatal dopamine levels in MPTP-treated mice

**Fig 4:**
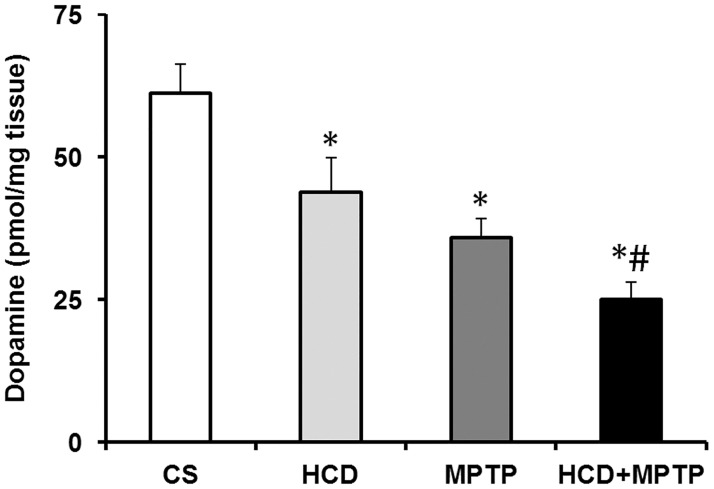
Effect of hypercholesterolemia on striatal dopamine level in Parkinsonian mice. Mice were sacrificed by decapitation on the seventh day following the first dose of MPTP. Striatal dopamine content was analyzed by HPLC-ECD system. Hypercholesterolemia exaggerates striatal dopamine depletion in Parkinsonian mice. The results are given as mean ± S.E.M. *p ≤ 0.05 as compared to control (CS) and #p ≤ 0.05 as compared to MPTP alone treated group (n = 6).

MPTP treatment caused a significant decrease in striatal dopamine level by 42% as compared to control (Fig 4). In striatum, dopamine levels were reduced by 28% in HCD mice, which differed significantly from the control group. Interestingly, cholesterol aggravated the loss of striatal dopamine levels in HCD+MPTP animals compared to MPTP alone treated animals. In HCD+MPTP animals, dopamine levels were significantly reduced by 58% as compared to control and 30% as compared to MPTP alone treated animals.

### Hypercholesterolemia on dopaminergic neurodegeneration in MPTP-treated mice

**Fig 5:**
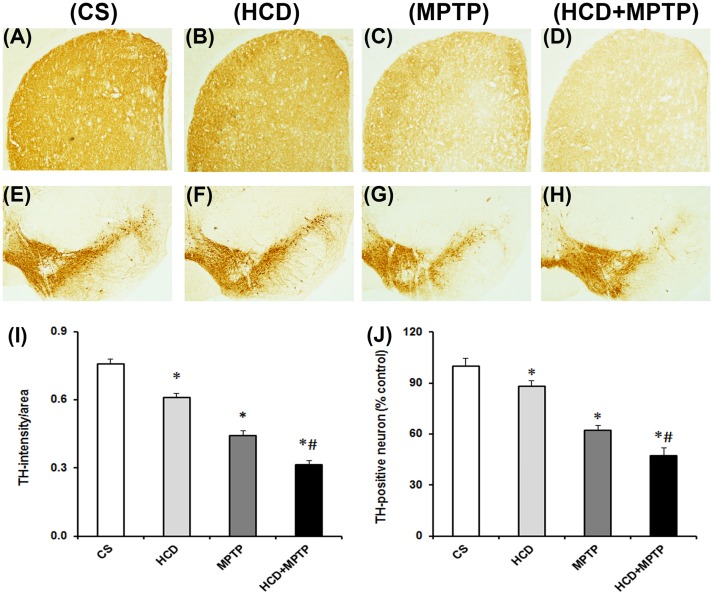
Effects of hypercholesterolemia on tyrosine hydroxylase (TH)-immunoreactivity in striatum (NCP) and TH-positive nigral (SN) neurons in MPTP-treated mice. Representative NCP (A-D) and SN (E-H) photographs from CS, HCD, MPTP and HCD+MPTP (left to right) groups showing TH-immunoreactivity. Quantification of relative density of TH-immunostaining in NCP (I) and neuronal count in SN (J) analyzed using ImageJ software. Hypercholesterolemia aggravates nigral TH-positive neuronal loss in MPTP-treated mice. Results are expressed as mean ± SEM. *p ≤ 0.05 as compared to control (CS) and #p ≤ 0.05 as compared to MPTP alone treated group (n = 5).

MPTP administration caused a significant loss of nigral dopaminergic neurons by 38% (Fig 5G) and the TH-immunoreactivity in striatum was reduced by 42% (Fig 5C) compared to the corresponding regions of control animals. Interestingly, there occurred a significant loss of TH-positive neurons in SN by 12% (Fig 5F) while the TH-immunoreactivity by 20% (Fig 5B) in animals that were subjected to HCD as compared to control. Moreover, MPTP administration in HCD mice caused a significant loss of TH-positive neurons in SN by 53% (Fig 5H) and striatal TH-immunoreactivity by 59% (Fig 5D) as compared to MPTP alone treated animals (Fig 5C and 5G). Thereby, hypercholesterolemia aggravated the nigral dopaminergic neurodegeneration in MPTP mouse model of PD.

### Hypercholesterolemia on nigrostriatal mitochondrial complexes in MPTP model of PD

**Fig 6:**
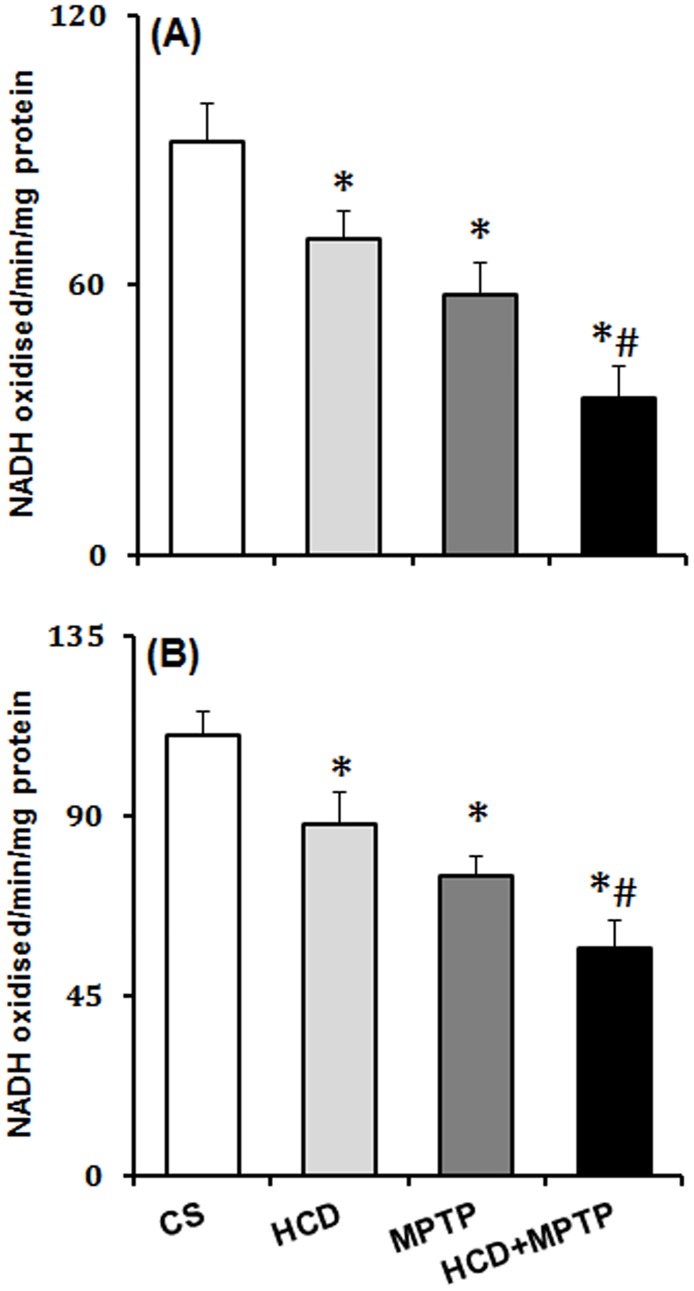
Effect of hypercholesterolemia on nigrostriatal mitochondrial complex-I activity in Parkinsonian mice. Mitochondrial complex-I activity was analyzed in the (A) striatum and (B) substantia nigra regions of brain by employing a spectrophotometric procedure using NADH as substrate. Results given are mean ± SEM of nmol of NADH oxidized/min/mg protein. *p ≤ 0.05 as compared to control (CS) and #p ≤ 0.05 as compared to MPTP alone treated group (n = 4).

In HCD mice and Parkinsonian mice, mitochondrial complex-I activity significantly inhibited by 23% and 36% in NCP (Fig 6A) and 20% and 31% in SN (Fig 6B) respectively, compared to the control animals. Moreover, in MPTP-treated HCD animals, complex-I activity was further reduced significantly by 39% in NCP and 24% in SN compared to the animals treated with MPTP only (Fig 6). Thus, cholesterol significantly potentiated MPTP-induced reduction of complex-I activity.

**Fig 7:**
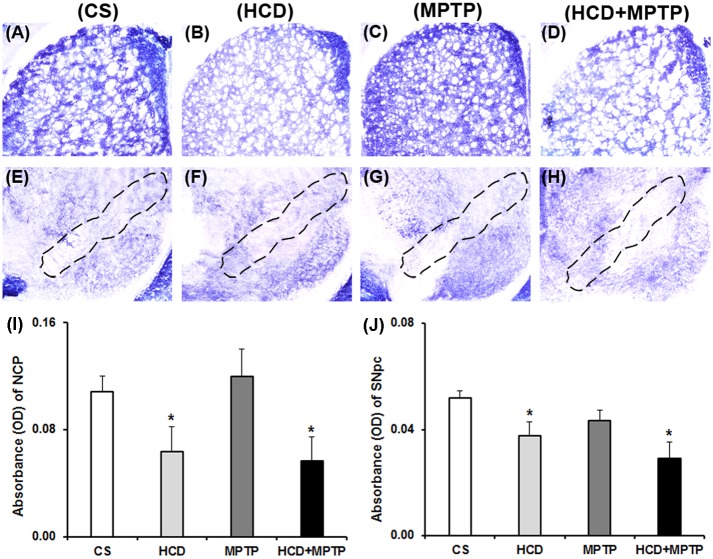
Effect of hypercholesterolemia on nigrostriatal mitochondrial complex-II activity in Parkinsonian mice. (A-D) representative striatal (NCP) sections and (E-H) substantia nigral (SN) sections were processed for mitochondrial complex-II activity by employing histoenzymology. The marked region in the photographs (E-H) represents substantia nigra pars compacta (SNpc) region [59]. Optical density of serial sections of (I) NCP and (J) SN was analyzed using ImageJ software. The results are given as mean ± S.E.M. *p ≤ 0.05 as compared to control (CS) and #p ≤ 0.05 as compared to MPTP alone treated group (n = 4).

**Fig 8:**
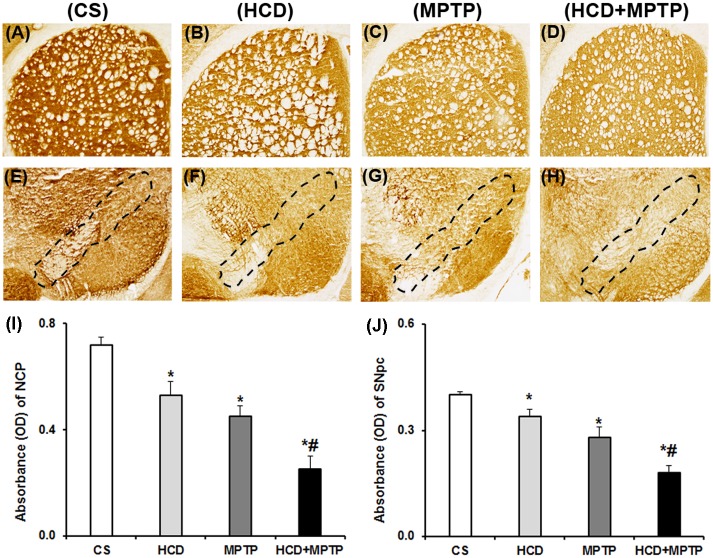
Effect of hypercholesterolemia on nigrostriatal mitochondrial complex-III activity in Parkinsonian mice. (A-D) representative striatal (NCP) sections and (E-H) substantia nigral (SN) sections were processed for mitochondrial complex-III activity by employing histoenzymology. The marked region in the photographs (E-H) represents substantia nigra pars compacta (SNpc) region [59]. Optical density of serial sections of (I) NCP and (J) SN was analyzed using ImageJ software. The results are given as mean ± S.E.M. *p ≤ 0.05 as compared to control (CS) and #p ≤ 0.05 as compared to MPTP alone treated group (n = 4).

MPTP administration did not affect the nigrostriatal mitochondrial complex-II activity in naïve animals. While in HCD animals, the activity of complex-II was reduced significantly by 41% in NCP and 27% in SN as compared to the control (Fig 7). Cholesterol did not cause any significant change in the activity of complex-II in HCD+MPTP animals compared to MPTP-treated animals. Histochemical investigation of mitochondrial complex-III activity revealed a marked visible decrease in the enzyme activity in nigrostriatal pathway of both HCD and MPTP-treated animals compared to the control (Fig 8). The staining intensity of complex-III activity was reduced significantly by 27%, 36% and 65% in NCP (Fig 8B, 8C and 8D), while 15%, 29% and 54% in SN of HCD, MPTP and HCD+MPTP mice respectively (Fig 8F, 8G and 8H) compared to the control. Most importantly, administration of MPTP in HCD animals significantly potentiated MPTP-induced reduction of complex-III activity by 44% in NCP and 36% in SN, compared to MPTP-treated animals (Fig 8I and 8J).

### Hypercholesterolemia on nigrostriatal hydroxyl radical levels in MPTP model of PD

**Fig 9:**
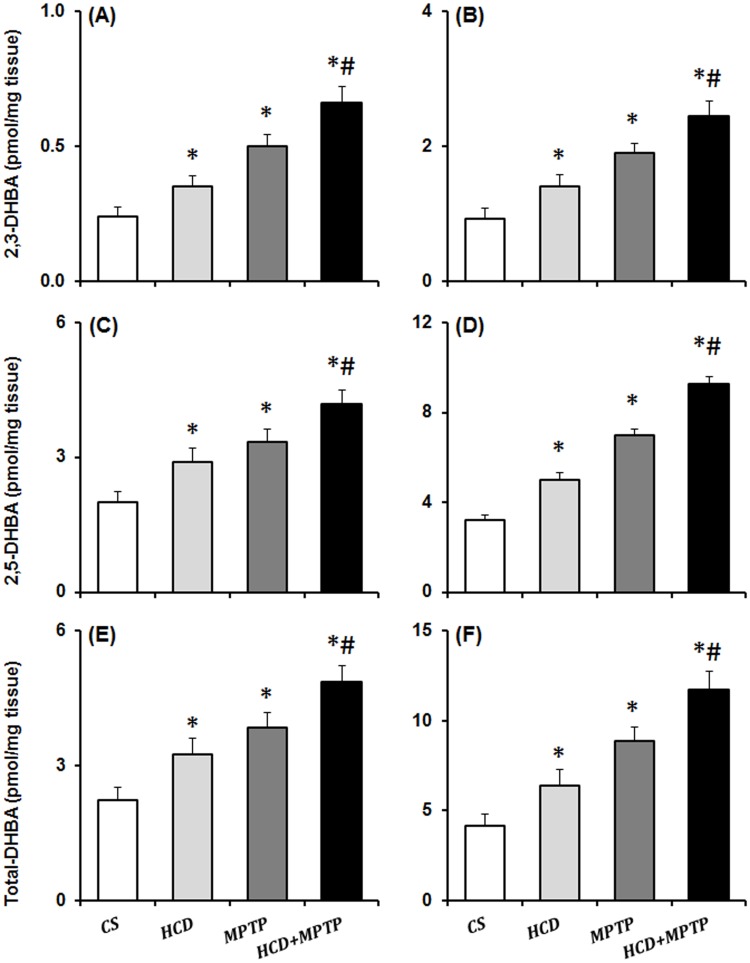
Effect of hypercholesterolemia on hydroxyl radical (•OH) generation in (A,C,E) striatum and (B,D,F) substantia nigra regions of brain of Parkinsonian mice. Animals were injected with salicylic acid (100 mg/kg) and sacrificed two hours post injection on the last day of treatment. 2,3- and 2,5-dihydroxy benzoic acid (DHBA; •OH adducts of salicylate) formed were measured from homogenates of NCP and SN by employing a sensitive HPLC-ECD method. Data are expressed as pmol/mg tissue and represented as mean ± S.E.M. *p ≤ 0.05 as compared with control and #p ≤ 0.05 as compared with MPTP alone treated group (n = 5).

HCD caused a significant increase in the levels of •OH in the nigrostriatal pathway as revealed from increased levels of 2,3- and 2,5-DHBA (Fig 9). In HCD animals, the levels of 2,3-DHBA and 2,5-DHBA were increased by 1.46- and 1.45-fold in NCP (Fig 9A and 9C), and 1.51- and 1.56-fold in SN (Fig 9B and 9D) respectively as compared to controls. In NCP and SN, the levels of 2,3-DHBA were significantly increased by 1.32- and 1.29-fold respectively, and the levels of 2,5-DHBA were significantly increased by 1.25- and 1.33-fold respectively in HCD+MPTP animals compared to MPTP alone treated animals (Fig 9). Thus, in HCD+MPTP animals, cholesterol exaggerated the MPTP-induced generation of DHBA in the nigrostriatal pathway.

### Hypercholesterolemia on nigrostriatal glutathione levels in MPTP model of PD

**Fig 10:**
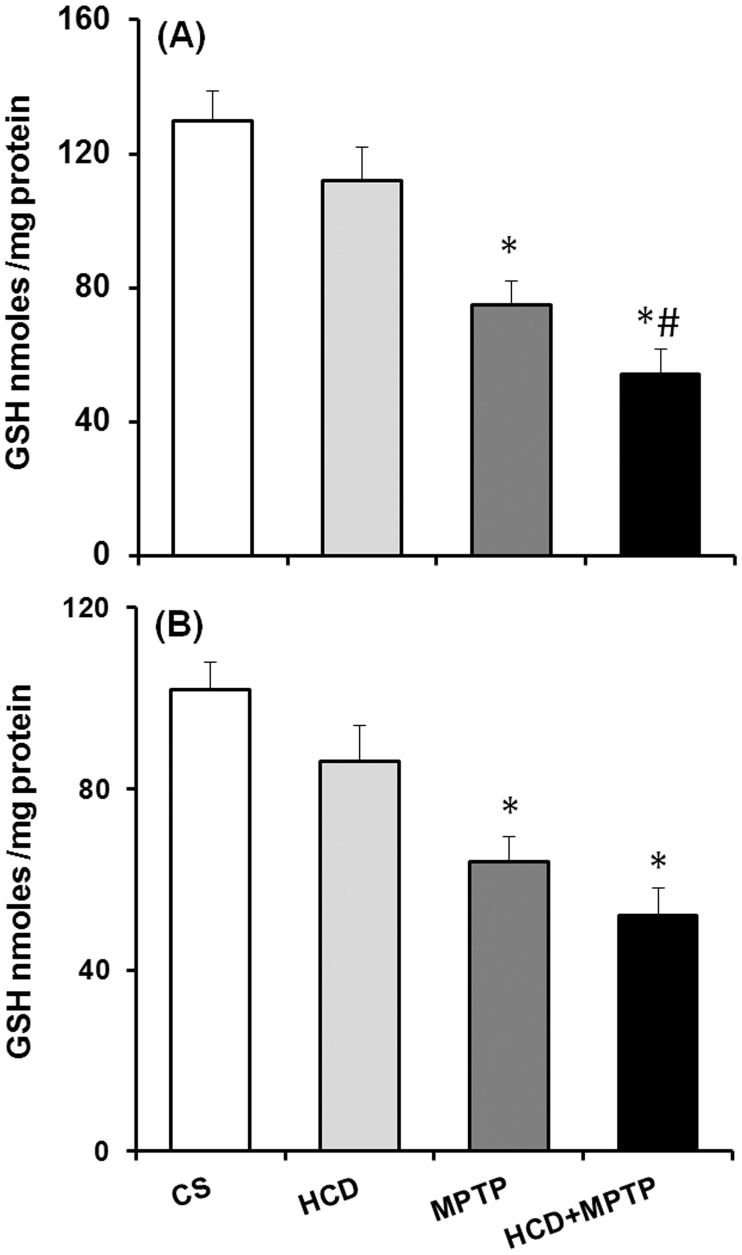
Effect of hypercholesterolemia on nigrostriatal reduced glutathione (GSH) level in Parkinsonian mice. (A) Striatum and (B) substantia nigra regions of brain were used for estimating GSH levels by employing a sensitive HPLC-ECD system. Data are expressed as nmol/mg tissue and represented as mean ± S.E.M. *p ≤ 0.05 as compared with control and #p ≤ 0.05 as compared with MPTP alone treated group (n = 5).

HCD for 14-weeks did not cause any significant alteration in the levels of GSH in the nigrostriatal pathway as compared to the controls (Fig 10). In animals treated with MPTP alone, GSH levels were decreased significantly by 42% in NCP and 37% in SN as compared to the controls. While a significant decrease in GSH level was found in both NCP (by 58%) and SN (by 49%) in HCD+MPTP animals as compared to control. Interestingly, in HCD+MPTP animals, hypercholesterolemia significantly increased MPTP-induced GSH depletion in NCP by 28% (Fig 10A), but not in the SN region of the brain (Fig 10B).

### Hypercholesterolemia on antioxidant enzyme activity in MPTP model of PD

**Fig 11:**
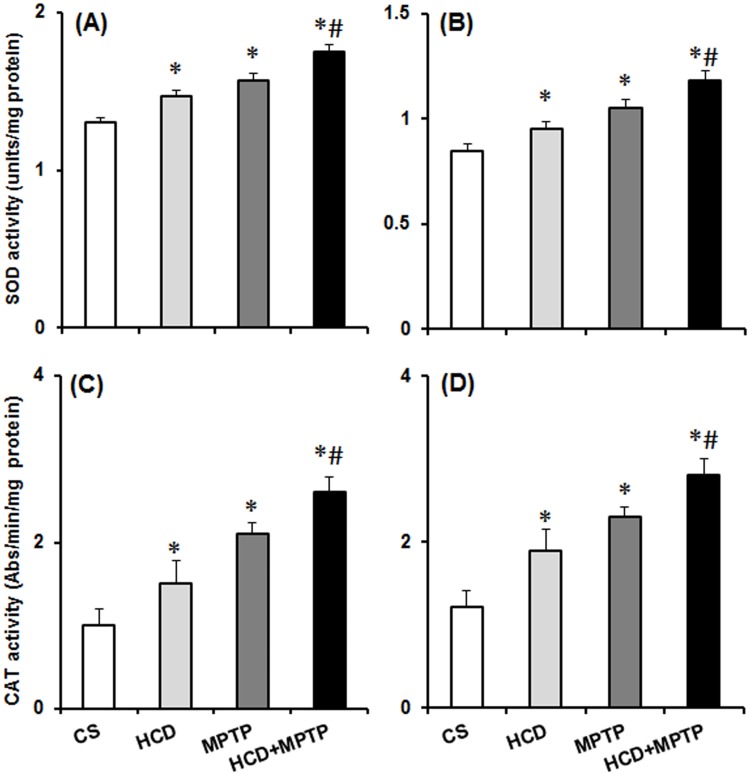
Effect of hypercholesterolemia on nigrostriatal antioxidant enzymes activity in Parkinsonian mice. (A-B) superoxide dismutase (SOD) and (C-D) catalase (CAT) activity measured from the cytosolic fraction of (A) striatum and (B) substantia nigra regions of brain. SOD activity was analyzed by employing pyrogallol oxidation method. One unit of the SOD activity is defined as 50% inhibition/min/mg protein. CAT activity was analyzed by monitoring the disappearance of hydrogen peroxide in presence of the enzyme. Specific activity of CAT is described as change in absorbance at 240 nm/min/mg protein. The results are given as mean ± S.E.M. *p ≤ 0.05 as compared to control (CS) and #p ≤ 0.05 as compared to MPTP alone treated group (n = 5).

The activity of antioxidant enzymes (SOD and catalase) was analyzed from the cytosolic fraction of NCP and SN regions of the brain (Fig 11). In HCD as well as Parkinsonian mice, nigrostriatal SOD and catalase activity was elevated significantly as compared to the control animals. In NCP and SN, 1.13- and 1.12-fold increase in SOD activity was observed (Fig 11A and 11B), whereas catalase activity increased by 1.5- and 1.56-fold respectively in HCD mice (Fig 11C and 11D) compared to the controls. MPTP treatment caused 1.21- and 1.24-fold increase in SOD activity, whereas catalase activity was increased by 2.1- and 1.89-fold respectively in the NCP and SN regions as compared to the controls. Compared to the Parkinsonian mice, SOD activity was significantly elevated both in NCP (by 1.1-fold) and SN (by 1.12-fold) in HCD+MPTP mice. Catalase activity was further elevated by 1.24-fold in NCP and 1.22-fold in SN of HCD+MPTP mice compared to MPTP alone treated animals (Fig 11). Thus, MPTP-induced oxidative stress in dopaminergic neurons was further potentiated in hypercholesterolemic condition.

## Discussion

Findings from the present study suggests for the involvement of hypercholesterolemia in nigral dopaminergic neurodegeneration, which is aggravated in MPTP model of PD *via* mitochondrial dysfunction and oxidative stress. The key findings of the present study are that hypercholesterolemia in MPTP model of PD (i) worsens Parkinsonian motor behavior, (ii) exaggerates striatal dopamine loss and nigral dopaminergic neurodegeneration, (iii) reduces mitochondrial complexes (I and III) activity in the nigrostriatal pathway, and (iv) intensifies oxidative stress in the nigrostriatal pathway.

The significant increase in body weight in animals subjected to HCD from the 4^th^ to 8^th^ weeks followed by no significant change is a symptom of hypercholesterolemic model [Fig 2A; 41,42]. The levels of serum cholesterol level in HCD animals were found to increase by 2-fold (Fig 2B), which confirms hypercholesterolemia in animals at biochemical level [41,43]. Moreover, HCD resulted in a significant accumulation of cholesterol in liver (Fig 2D) and in dopamine-rich region of brain (NCP; Fig 2F**)**, which further consolidated the hypercholesterolemic model of the present study. Mice that were administered with MPTP (30 mg/kg; 2 doses) displayed motor abnormalities (Fig 3) with significant loss of dopamine in NCP (by 42%; Fig 4) and TH-positive neurons in SN (by 38%; Fig 5), which are consistent with the previous studies of PD induced by MPTP [30,44].

Although case-control studies have provided a strong association of cholesterol levels and occurrence of PD [13,14], no such study has been performed in animal models and thus the underlying mechanism remains largely elusive. Studies reported that high-fat diet for 8-weeks exacerbates Parkinsonian neurotoxin (MPTP and 6-hydroxydopamine) induced striatal dopamine depletion with degeneration of dopaminergic axonal terminals in striatum in mice [15,16]. However, the direct effect of cholesterol on nigral dopaminergic neurons was largely limited from the previous studies [15,16]. High-fat diet is widely used to generate obese as well as hypercholesterolemic animals. However, the greater amount of saturated fatty acid in high-fat diet has been reported to cause cognitive impairments, dementia and neurodegenerative like pathology in animal models [45–47]. Moreover, high fat diet causes insulin resistance which has been reported to have adverse effects on brain functions [48,49]. Therefore, high cholesterol diet (5% cholesterol mixed with normal rodent chow) was used in the present study for inducing hypercholesterolemia which is a novel approach (Fig 1). Our result that hypercholesterolemia causes nigral dopaminergic neurodegeneration with concomitant depletion of striatal dopamine, and exacerbates dopaminergic neurodegeneration in Parkinsonian mice, is the first report of such kind. Thus, the results provide experimental direct evidence of neurotoxic potential of cholesterol on dopaminergic neurons.

The loss of significant motor activity in hypercholesterolemic animals is similar to that found in experimental models of PD [28,30,50]. The display of increased latency in akinesia and catalepsy and decrease in swimming ability are indications of reduced motor activity (Fig 3). An important factor in the appearance of Parkinsonian motor symptom is the loss of striatal dopamine [28,30,44,51]. The relatively small depletion of striatal dopamine is associated with the loss of striatal TH-immunoreactivity and nigral dopaminergic neurons in hypercholesterolemic mice. Thus, the observed equivalent Parkinsonian motor symptoms in the hypercholesterolemic mice might be linked with the loss of striatal dopamine. Interestingly, the proximity of Parkinsonian motor symptoms was found to be severely affected in hypercholesterolemic mice treated with MPTP (Fig 3), where the striatal dopamine levels were depleted to a greater extent compared to control as well as Parkinsonian mice (Fig 4). Thus, the result implies the aggravation of Parkinsonian symptoms by hypercholesterolemia.

Altered mitochondrial complexes function and subsequent generation of free radical-induced oxidative stress is regarded as underlying events in PD pathology [5–7]. In PD patients, mitochondrial complex-I activity has been found to decrease in the SN, striatum and frontal cortex [52,53]. Postmortem studies indicated down-regulation of antioxidant defense mechanisms in PD brain, including GSH, SOD, and catalase [54,55]. Therefore, the status of mitochondrial complexes and antioxidant defense has been investigated to unveil the underlying mechanism of hypercholesterolemia-induced dopaminergic neurotoxicity in the mice model of PD. There was a severe impairment of mitochondrial complexes (I, II and III) activity in nigrostriatal pathway of hypercholesterolemic animals. Moreover, hypercholesterolemia significantly increased the impairment of mitochondrial complexes activity, mainly complex-I (Fig 6) and complex-III (Fig 8) in Parkinsonian mice. The level of •OH radicals (Fig 9) and the activity of antioxidant enzymes, SOD and catalase (Fig 11), were found to be elevated significantly in the nigrostriatal pathway of hypercholesterolemic animals treated with or without MPTP. However, no significant alteration in the levels of endogenous antioxidant molecule (GSH) was observed in hypercholesterolemic animals (Fig 10). However, compared to the Parkinsonian mice, hypercholesterolemia significantly contributed to decrease in GSH levels in NCP caused by MPTP. Alteration in the activity of antioxidant enzymes and decrease in GSH levels has been reported previously in non-dopaminergic brain regions of hypercholesterolemic animals [20–23]. Several reports provided evidence of oxidative stress as a result of elevated levels •OH radicals and antioxidant enzymes with concomitant loss of GSH, and complex-I inhibition resulting causing midbrain dopaminergic neuronal loss in the MPTP model of PD [34,44,56,57]. Moreover, increased oxidative stress known to damage the functioning of mitochondrial complexes with a rise in generation of free radicals, which further hampers the cellular antioxidant defense homeostasis [6,58]. Thus, it may be argued that hypercholesterolemia-induced increased level •OH and enhanced activity of antioxidant enzymes in the nigrostriatal pathway leads to oxidative stress and mitochondrial dysfunction, which together exaggerates the Parkinsonian pathology.

The present study is the first direct *in vivo* evidence of hypercholesterolemia-induced midbrain dopaminergic neurodegeneration and exacerbation of Parkinsonian symptoms as well as signature pathologies in the MPTP model of PD. Our results further demonstrate the involvement of mitochondrial dysfunctions and oxidative stress or *vice versa* in hypercholesterolemia-induced dopaminergic neurotoxicity.

## References

[1] Pan-Montojo F, Anichtchik O, Dening Y, Knels L, Pursche S, Jung R, et al Progression of Parkinson's disease pathology is reproduced by intragastric administration of rotenone in mice. PLoS One. 2010;5(1): e8762 10.1371/journal.pone.0008762 20098733 PMC2808242

[2] Kalia LV, Lang AE. Parkinson’s disease. Lancet. 2015;386(9996): 896–912. 10.1016/S0140-6736(14)61393-3 25904081

[3] Di Maio R, Barrett PJ, Hoffman EK, Barrett CW, Zharikov A, Borah A, et al alpha-Synuclein binds to TOM20 and inhibits mitochondrial protein import in Parkinson’s disease. Sci Transl Med. 2016;8(6): 342ra78. 10.1126/scitranslmed.aaf3634 PMC5016095 27280685

[4] Mazzoni P, Shabbott B, Cortés JC. Motor control abnormalities in Parkinson’s disease. Cold Spring Harb Perspect Med. 2012;2(6):a009282 10.1101/cshperspect.a009282 22675667 PMC3367543

[5] Panov A, Dikalov S, Shalbuyeva N, Taylor G, Sherer T, Greenamyre JT. Rotenone model of Parkinson disease: Multiple brain mitochondria dysfunctions after short term systemic rotenone intoxication. J Biol Chem. 2005;280(51): 42026–42035. 10.1074/jbc.M508628200 16243845

[6] Dexter DT, Jenner P. Parkinson disease: from pathology to molecular disease mechanisms. Free Radic Biol Med. 2013;62: 132–144. 10.1016/j.freeradbiomed.2013.01.018 23380027

[7] Medeiros MS, Schumacher-Schuh A, Cardoso AM, Bochi GV, Baldissarelli J, Kegler A, et al Iron and Oxidative Stress in Parkinson's Disease: An Observational Study of Injury Biomarkers. PLoS One. 2016;11(1): e0146129 10.1371/journal.pone.0146129 26751079 PMC4709097

[8] Borah A, Mohanakumar KP. L-DOPA-induced 6-hydroxydopamine production in the striata of rodents is sensitive to the degree of denervation. Neurochem Int. 2010;56(2): 357–362. 10.1016/j.neuint.2009.11.008 19931335

[9] Mazumder MK, Paul R, Borah A. β-Phenethylamine-A phenylalanine derivative in brain-contributes to oxidative stress by inhibiting mitochondrial complexes and DT-diaphorase: An in silico study. CNS Neurosci Ther. 2013;19(8):596–602. 10.1111/cns.12113 23638910 PMC6493350

[10] Paul R, Borah A. L-DOPA-induced hyperhomocysteinemia in Parkinson's disease: Elephant in the room. Biochim Biophys Acta. 2016;1860(9): 1989–1997. 10.1016/j.bbagen.2016.06.018 27318154

[11] Paul R, Choudhury A, Borah A. Cholesterol—A putative endogenous contributor towards Parkinson’s disease. Neurochem Int. 2015;90(11): 125–133. 26232622 10.1016/j.neuint.2015.07.025

[12] Doria M, Maugest L, Moreau T, Lizard G, Vejux A. Contribution of cholesterol and oxysterols to the pathophysiology of Parkinson's disease. Free Radic Biol Med. 2016;101: 393–400. 10.1016/j.freeradbiomed.2016.10.008 27836779

[13] Hu G, Antikainen R, Jousilahti P, Kivipelto M, Tuomilehto J. Total cholesterol and the risk of Parkinson disease. Neurology. 2008;70(21): 1972–1979. 10.1212/01.wnl.0000312511.62699.a8 18401018

[14] Hu G, Jousilahti P, Nissinen A, Antikainen R, Kivipelto M, Tuomilehto J. Body mass index and the risk of Parkinson disease. Neurology. 2006;67(11): 1955–1959. 10.1212/01.wnl.0000247052.18422.e5 17159100

[15] Bousquet M, St-Amour I, Vandal M, Julien P, Cicchetti F, Calon F. High-fat diet exacerbates MPTP-induced dopaminergic degeneration in mice. Neurobiol Dis. 2012; 45(1): 529–538. 10.1016/j.nbd.2011.09.009 21971528

[16] Choi JY, Jang EH, Park CS, Kang JH. Enhanced susceptibility to 1-methyl-4-phenyl-1,2,3,6-tetrahydropyridine neurotoxicity in high-fat diet-induced obesity. Free Radic Biol Med. 2005;38(6): 806–816. 10.1016/j.freeradbiomed.2004.12.008 15721991

[17] Bar-On P, Crews L, Koob AO, Mizuno H, Adame A, Spencer B, et al Statins reduce neuronal alpha-synuclein aggregation in in vitro models of Parkinson’s disease. J Neurochem. 2008;105(5): 1656–1667. 10.1111/j.1471-4159.2008.05254.x 18248604 PMC2822545

[18] Marwarha G, Rhen T, Schommer T, Ghribi O. The oxysterol 27-hydroxycholesterol regulates α-synuclein and tyrosine hydroxylase expression levels in human neuroblastoma cells through modulation of liver X receptors and estrogen receptors-relevance to Parkinson’s disease. J Neurochem. 2011;119(5): 1119–1113. 10.1111/j.1471-4159.2011.07497.x 21951066 PMC3217121

[19] Rantham Prabhakara JP, Feist G, Thomasson S, Thompson A, Schommer E, Ghribi O. Differential effects of 24-hydroxycholesterol and 27-hydroxycholesterol on tyrosine hydroxylase and alpha-synuclein in human neuroblastoma SH-SY5Y cells. J Neurochem. 2008;107(6): 1722–1729. 10.1111/j.1471-4159.2008.05736.x 19014385

[20] Aytan N, Jung T, Tamtürk F, Grune T, Kartal-Ozer N. Oxidative stress related changes in the brain of hypercholesterolemic rabbits. Biofactors. 2008;33(3): 225–236. 19478426 10.1002/biof.5520330308

[21] Prasanthi JRP, Dasari B, Marwarha G, Larson T, Chen X, Geiger JD, et al Caffeine protects against oxidative stress and Alzheimer’s disease-like pathology in rabbit hippocampus induced by cholesterol-enriched diet. Free Radic Biol Med. 2010;49: 1212–1220. 10.1016/j.freeradbiomed.2010.07.007 20638472 PMC2930139

[22] de Oliveira J, Hort MA, Moreira EL, Glaser V, Ribeiro-do-Valle RM, Prediger RD, et al Positive correlation between elevated plasma cholesterol levels and cognitive impairments in LDL receptor knockout mice: relevance of cortico-cerebral mitochondrial dysfunction and oxidative stress. Neuroscience. 2011;197: 99–106. 10.1016/j.neuroscience.2011.09.009 21945034

[23] de Oliveira J, Moreira ELG, Mancini G, Hort MA, Latini A, Ribeiro-Do-Valle RM, et al Diphenyl diselenide prevents cortico-cerebral mitochondrial dysfunction and oxidative stress induced by hypercholesterolemia in LDL receptor knockout mice. Neurochem Res. 2013;38: 2028–2036. 10.1007/s11064-013-1110-4 23881289

[24] Allain CC, Poon LS, Chan CSG, Richmond W, Fu PC. Enzymatic determination of total serum cholesterol. Clin Chem. 1974;20: 470–475. 4818200

[25] Weber AF, Phillips MG, Bell JTJ. An improved method for the Schultz cholesterol test. J Histochem Cytochem. 1956;4: 308–309. 13346060 10.1177/4.4.308

[26] Schindelin J, Arganda-Carreras I, Frise E, Kaynig V, Longair M, Pietzsch T, et al Fiji: an open-source platform for biological-image analysis. Nat Methods. 2012;9: 676–682. 10.1038/nmeth.2019 22743772 PMC3855844

[27] Mazumder MK, Giri A, Kumar S, Borah A. A highly reproducible micemodel of chronic kidney disease: Evidences of behavioural abnormalities and blood-brain barrier disruption. Life Sciences. 2016;161: 27–36. 10.1016/j.lfs.2016.07.020 27493078

[28] Haobam R, Sindhu KM, Chandra G, Mohanakumar KP. Swim-test as a function of motor impairment in MPTP model of Parkinson’s disease: A comparative study in two mouse strains. Behav Brain Res. 2005;163: 159–167. 10.1016/j.bbr.2005.04.011 15941598

[29] Borah A, Mohanakumar KP. Long-term L-DOPA treatment causes indiscriminate increase in dopamine levels at the cost of serotonin synthesis in discrete brain regions of rats. Cell Mol Neurobiol. 2007;27(8): 985–996. 10.1007/s10571-007-9213-6 17934805 PMC11517132

[30] Bhattacharjee N, Mazumder MK, Paul R, Choudhury A, Choudhury S, Borah A. L-DOPA treatment in MPTP-mouse model of Parkinson’s disease potentiates homocysteine accumulation in substantia nigra. Neurosci Lett. 2016;628: 225–229. 10.1016/j.neulet.2016.06.011 27283777

[31] Karuppagounder SS, Madathil SK, Pandey M, Haobam R, Rajamma U, Mohanakumar KP. Quercetin up-regulates mitochondrial complex-I activity to protect against programmed cell death in rotenone model of Parkinson’s disease in rats. Neuroscience. 2013;236: 136–148. 10.1016/j.neuroscience.2013.01.032 23357119

[32] Pandey M, Varghese M, Sindhu KM, Sreetama S, Navneet AK, Mohanakumar KP, et al Mitochondrial NAD+-linked State 3 respiration and complex-I activity are compromised in the cerebral cortex of 3-nitropropionic acid-induced rat model of Huntington’s disease. J Neurochem. 2008;104: 420–434. 10.1111/j.1471-4159.2007.04996.x 17953654

[33] Govindaiah, Shankaranarayana Rao BS, Ramamohan Y, Singh YK, Dhingra NK, Raju TR. Cytochrome oxidase activity in rat retinal ganglion cells during postnatal development. Brain Res Dev Brain Res. 2000;124: 117–120. 11113518 10.1016/s0165-3806(00)00092-4

[34] Thomas B, Mohanakumar KP. Melatonin protects against oxidative stress caused by 1-methyl-4-phenyl-1,2,3,6-tetrahydropyridine in the mouse nigrostriatum. J Pineal Res. 2004;36(1): 25–32. 14675127 10.1046/j.1600-079x.2003.00096.x

[35] Melnyk S, Pogribna M, Pogribny I, Hine RJ, James SJ. A new HPLC method for the simultaneous determination of oxidized and reduced plasma aminothiols using coulometric electrochemical detection. J Nutr Biochem. 1999;10: 490–497. 15539328 10.1016/s0955-2863(99)00033-9

[36] Marklund S, Marklund G. Involvement of the superoxide anion radical in the autoxidation of pyrogallol and a convenient assay for superoxide dismutase. Eur J Biochem. 1974;47: 469–474. 4215654 10.1111/j.1432-1033.1974.tb03714.x

[37] Bhattacharjee N, Paul R, Giri A, Borah A. Chronic exposure of homocysteine in mice contributes to dopamine loss by enhancing oxidative stress in nigrostriatum and produces behavioral phenotypes of Parkinson’s disease. Biochem Biophys Rep. 2016;6: 47–53. 28955861 10.1016/j.bbrep.2016.02.013 PMC5600271

[38] Aebi H. Catalase in vitro. Methods Enzymol. 1984;105: 121–126. 6727660 10.1016/s0076-6879(84)05016-3

[39] Tripathy D, Verma P, Nthenge-Ngumbau DN, Banerjee M, Mohanakumar KP. Regenerative therapy in experimental parkinsonism: mixed population of differentiated mouse embryonic stem cells, rather than magnetically sorted and enriched dopaminergic cells provide neuroprotection. CNS Neurosci Ther. 2014;20: 717–727. 10.1111/cns.12295 24954161 PMC6493138

[40] Tapias V, Cannon JR, Greenamyre JT. Melatonin treatment potentiates neurodegeneration in a rat rotenone Parkinson’s disease model. J Neurosci Res. 2010;88: 420–427. 10.1002/jnr.22201 19681169

[41] Ullrich C, Pirchl M, Humpel C. Hypercholesterolemia in rats impairs the cholinergic system and leads to memory deficits. Mol Cell Neurosci. 2010;45(4): 408–417. 10.1016/j.mcn.2010.08.001 20696249 PMC2977849

[42] Rao W, Su Y, Yang G, Ma Y, Liu R, Zhang S, Wang S, Fu Y, Kou C, Yu Y, Yu Q. Cross-Sectional Associations between Body Mass Index and Hyperlipidemia among Adults in Northeastern China. Int J Environ Res Public Health. 2016;13(5): 516. 10.3390/ijerph13050516 PMC4881141 27213419

[43] Refolo LM, Malester B, LaFrancois J, Bryant-Thomas T, Wang R, Tint GS, et al Hypercholesterolemia accelerates the Alzheimer's amyloid pathology in a transgenic mouse model. Neurobiol Dis. 2000;7(4): 321–331. 10.1006/nbdi.2000.0304 10964604

[44] Naskar A, Prabhakar V, Singh R, Dutta D, Mohanakumar KP. Melatonin enhances L-DOPA therapeutic effects, helps to reduce its dose, and protects dopaminergic neurons in 1-methyl-4-phenyl-1,2,3,6-tetrahydropyridine-induced Parkinsonism in mice. J Pineal Res. 2015;58(3): 262–274. 10.1111/jpi.12212 25626558

[45] Knight EM, Martins IV, Gümüsgöz S, Allan SM, Lawrence CB. High-fat diet-induced memory impairment in triple-transgenic Alzheimer's disease (3xTgAD) mice is independent of changes in amyloid and tau pathology. Neurobiol Aging. 2014;35(8): 1821–1832. 10.1016/j.neurobiolaging.2014.02.010 24630364 PMC4024197

[46] Toyama K, Koibuchi N, Hasegawa Y, Uekawa K, Yasuda O, Sueta D, et al ASK1 is involved in cognitive impairment caused by long-term high-fat diet feeding in mice. Sci Rep. 2015;5: 10844 10.1038/srep10844 26044555 PMC5377457

[47] Rabot S, Membrez M, Blancher F, Berger B, Moine D, Krause L, et al High fat diet drives obesity regardless the composition of gut microbiota in mice. Sci Rep. 2016;6: 32484 10.1038/srep32484 27577172 PMC5006052

[48] Hancock CR, Han DH, Chen M, Terada S, Yasuda T, Wright DC, et al High-fat diets cause insulin resistance despite an increase in muscle mitochondria. Proc Natl Acad Sci U S A. 2008;105(22): 7815–7820. 10.1073/pnas.0802057105 18509063 PMC2409421

[49] Zhu C, Schwarz P, Abakumova I, Aguzzi A. Unaltered Prion Pathogenesis in a Mouse Model of High-Fat Diet-Induced Insulin Resistance. PLoS One. 2015;10(12): e0144983 10.1371/journal.pone.0144983 26658276 PMC4677814

[50] Taylor TN, Greene JG, Miller GW. Behavioural phenotyping of mouse models of Parkinson's disease. Behav Brain Res. 2010;211(1): 1–10. 10.1016/j.bbr.2010.03.004 20211655 PMC2862121

[51] Sindhu KM, Banerjee R, Senthilkumar KS, Saravanan KS, Raju BC, Rao JM, et al Rats with unilateral median forebrain bundle, but not striatal or nigral, lesions by the neurotoxins MPP+ or rotenone display differential sensitivity to amphetamine and apomorphine. Pharmacol Biochem Behav. 2006;84(2): 321–329. 10.1016/j.pbb.2006.05.017 16820197

[52] Schapira AH, Cooper JM, Dexter D, Clark JB, Jenner P, Marsden CD, et al Mitochondrial complex I deficiency in Parkinson’s disease. Lancet. 1989;1(8649): 1269 2566813 10.1016/s0140-6736(89)92366-0

[53] Parker WD, Parks JK, Swerdlow RH. Complex I deficiency in Parkinson’s disease frontal cortex. Brain Res. 2008;1189: 215–218. 10.1016/j.brainres.2007.10.061 18061150 PMC2295283

[54] Ambani LM, Van Woert MH, Murphy S. Brain peroxidase and catalase in Parkinson disease. Arch Neurol. 1975;32(2):114–8. 1122174 10.1001/archneur.1975.00490440064010

[55] Pearce RKB, Owen A, Daniel S, Jenner P, Marsden CD. Alterations in the distribution of glutathione in the substantia nigra in Parkinson’s disease. J Neural Transm. 1997;104: 661–677. 10.1007/BF01291884 9444566

[56] Przedborski S, Jackson-Lewis V. Mechanisms of MPTP toxicity. Mov Disord. 1998;13 Suppl 1: 35–38. 9613716

[57] Meredith GE, Rademacher DJ. MPTP mouse models of Parkinson's disease: an update. J Parkinsons Dis. 2011;1(1): 19–33. 10.3233/JPD-2011-11023 23275799 PMC3530193

[58] Lin MT, Beal MF. Mitochondrial dysfunction and oxidative stress in neurodegenerative diseases. Nature. 2006;443: 787–795. 10.1038/nature05292 17051205

[59] Wey MC-Y, Fernandez E, Martinez PA, Sullivan P, Goldstein DS, et al neurodegeneration and motor dysfunction in mice lacking cytosolic and mitochondrial aldehyde dehydrogenases: Implications for Parkinson’s disease. PLoS One. 2012;7(2): e31522 10.1371/journal.pone.0031522 22384032 PMC3284575

